# Ground Moving Target 2-D Velocity Estimation and Refocusing for Multichannel Maneuvering SAR with Fixed Acceleration

**DOI:** 10.3390/s19173695

**Published:** 2019-08-25

**Authors:** Xinxin Tang, Xiaoling Zhang, Jun Shi, Shunjun Wei, Bokun Tian

**Affiliations:** School of Information and Communication Engineering, University of Electronic Science and Technology of China, No.2006, Xiyuan Ave, West Hi-Tech Zone, Chengdu 611731, China

**Keywords:** multichannel maneuvering synthetic aperture radar, ground moving target, 2D velocity estimation and refocusing, back projection, velocity SAR, velocity-aided BP, minimum entropy

## Abstract

It is difficult for multichannel maneuvering synthetic aperture radar (SAR) to achieve ground moving target 2D velocity estimation and refocusing. In this paper, a novel method based on back projection (BP) and velocity SAR (VSAR) is proposed to cope with the issues. First, the static scene is reconstructed by BP to solve the imaging problem of multichannel maneuvering SAR. Then, the static clutter is suppressed, and the range velocity is estimated via VSAR processing. As for azimuth velocity estimation and refocusing, a velocity search method based on velocity-aided BP (VA-BP) and VSAR is proposed to accomplish them. First, each azimuth velocity in the search and the estimated range velocity are used to image the moving target in a small-sized subimage space by VA-BP, i.e., matching the range history and the Doppler phase of the moving target in the image processing. Then, multiple sets of multichannel SAR subimages corresponding to different azimuth velocities are generated, and the clutter of each set of multichannel SAR subimages is also suppressed by VSAR processing. After that, the azimuth velocity is estimated by searching the clutter-suppressed subimage of the first spatial receiving channel in each set of multichannel SAR subimages with the best refocusing quality measured by the minimum entropy. Simulation results show the proposed method can reach high accuracy in moving target 2D velocity estimation and refocusing with the absolute error of 2D velocity estimation smaller than 0.1 m/s.

## 1. Introduction

Synthetic aperture radar (SAR) is an active microwave imaging sensor that can work independently of time and weather [1]. In recent years, ground moving target indication (GMTI), as one of the most important applications of SAR, has attracted great attention in civilian fields [2,3,4]. Currently, SAR-GMTI research is mainly focused on radar sensors mounted on platforms with a uniform linear trajectory. With the increasing demand for applications, more and more SAR systems are mounted on maneuvering platforms for land mapping, disaster monitoring, agriculture requirements, and so on. These platforms such as unmanned aerial vehicles [5,6], missiles [7] may have complex trajectories due to the existence of acceleration, which will bring a great challenge for moving target detection, imaging, and motion parameter estimation. Therefore, the study of ground moving target indication in a SAR with a maneuvering trajectory is of great significance.

GMTI can be performed with a single-channel SAR [8,9] or with a multichannel SAR [10,11]. The indication of moving targets for a single-channel SAR usually works well at a high signal-to-clutter ratio (SCR) [12]. With the increasing degree of spatial freedom, multichannel SAR can work well even at low SCR due to its strong clutter suppression ability. Conventional multichannel SAR-GMTI techniques include space time adaptive processing (STAP) [13], displacement phase center antenna (DPCA) [14,15], along-track interferometry (ATI) [16], and velocity SAR (VSAR) [17]. STAP has excellent performance for suppressing strong stationary clutter in theory. However, once the covariance matrix is not estimated precisely, the performance of clutter suppression will be degraded seriously, which results in a lower output SCR.

The DPCA technique eliminates the signals from stationary targets using a few along-track antennas [18]. Nevertheless, the traditional DPCA technique is limited by a strict relationship between pulse repetition frequency (PRF) and phase center interval. Although this limitation can be relaxed by interpolation in the image domain, it is also a problem to image well for maneuvering SAR using the traditional frequency domain imaging algorithm [19]. In addition, the DPCA technique cannot estimate the 2D velocity of the moving target.

ATI has the same antenna configuration as DPCA [20,21]. The difference is that ATI uses the phase shift between the along-track antennas, which is called the interferometry phase, to detect a moving target and estimate its radial velocity (i.e., line-of-sight). Unfortunately, the interferometry phase can be affected easily by the system noise, which will degrade the accuracy of detection and velocity estimation. Therefore, ATI is used in combination with DPCA (i.e., the DPCA-ATI method) for more accurate detection and velocity estimation [22]. However, the DPCA-ATI method can only estimate the radial velocity of the moving target, and it cannot estimate the azimuth velocity.

The VSAR technique is another multichannel SAR-GMTI technique, which suppresses the stationary clutter and estimates the radial velocity of the moving target by performing discrete Fourier transform (DFT) on the multiple complex images obtained from an along-track antenna array [23,24,25,26,27]. This technique has been experimentally tested by both ground-based and airborne multi-channel radar systems developed by the U.S. Naval Research Laboratory [28,29,30]. The attractiveness of the VSAR technique is its simplicity. However, the existing VSAR technique is based on the frequency domain imaging algorithm, which is difficult to apply directly in multichannel maneuvering SAR systems [31,32].

In this paper, a novel GMTI method based on back projection (BP) and VSAR is proposed to detect a moving target, estimate its 2D velocity, and refocus it for multichannel maneuvering SAR with fixed acceleration. First, the BP imaging model of a moving target is derived for multichannel maneuvering SAR to analyze the focus position and the peak response of the moving target in the image domain. Second, the VSAR method is utilized for moving target detection, range velocity estimation, and relocation. As for azimuth velocity estimation and refocusing, a velocity search method combining velocity-aided BP (VA-BP) and VSAR is proposed to accomplish them. First, the VA-BP imaging model of a moving target is derived for multichannel maneuvering SAR to analyze the focus position and the peak response of the moving target in the image domain, which provides a theoretical basis for the proposed azimuth velocity estimation and refocusing methods. Second, each azimuth velocity in the search and the estimated range velocity are used to image a moving target in a small-sized subimage space by VA-BP, i.e., matching the range history and the Doppler phase of the moving target in the image processing. Then, multiple sets of multichannel SAR subimages corresponding to different azimuth velocities are generated, and the clutter of each set of multichannel SAR subimages is suppressed by VSAR processing. After that, the azimuth velocity is estimated by searching the clutter-suppressed subimage of the first spatial receiving channel in each set of multichannel SAR subimages with the best refocusing quality measured by the minimum entropy. Since the velocity parameters are used for moving target imaging, the range migration and azimuth defocusing caused by the 2D velocity of the moving target can be eliminated, and the moving target will be focused well on its real position. Simulation results show that the proposed method can reach high accuracy in moving target 2D velocity estimation with the absolute error of 2D velocity estimation smaller than 0.1 m/s. Compared with the phase gradient autofocus (PGA) algorithm, the proposed method can achieve higher accuracy in moving target refocusing.

The remainder of this paper is arranged as follows. Section 2 gives the signal model of a moving target in the multichannel maneuvering SAR with fixed acceleration. Section 3 illustrates the proposed ground moving target 2D velocity estimation and refocusing method, and the numerical experiments are shown in Section 4. The last section concludes this paper.

## 2. Signal Model of a Moving Target in Multichannel Maneuvering SAR

**Figure 1:**
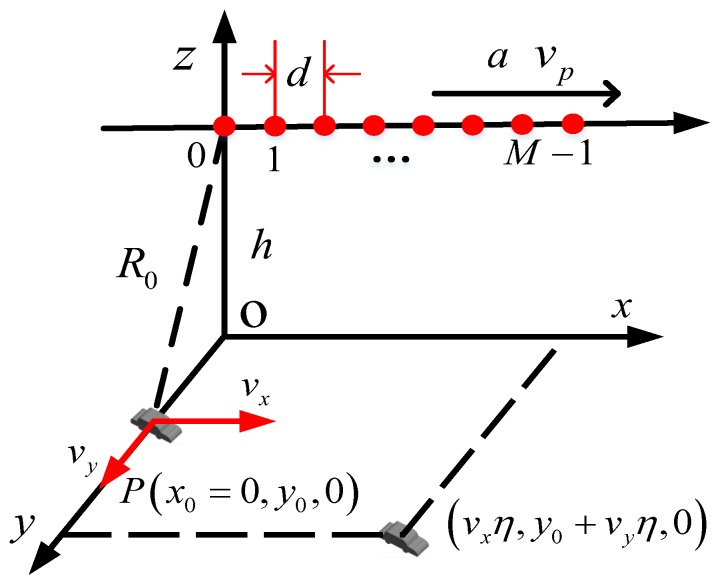
The geometry model of multichannel maneuvering SAR.

The geometry of multichannel maneuvering SAR with fixed acceleration is illustrated in Figure 1. The *x*-axis is the azimuth direction, and the *y*-axis is the range direction. The sampling time η along the *x*-axis is the slow time, while that of the *y*-axis, i.e., *t* is the fast time. The SAR system is equipped with a linear antenna array, including *M* receiving antenna elements with uniform space *d*. Only the zeroth antenna serves as both the transmitter and the receiver, while the others only serve as the receivers. The radar platform moves along the *x*-axis at altitude *h* with speed $v_{p}$ and constant acceleration *a*. When η=0, the $m^{\mathrm{th}}$ antenna element is located at (md,0,h) and a moving target $P_{MT}$ is located at $\left(x_{0}=0,y_{0}\right)$, where m=0,1,…,M−1. During the radar illumination, the target $P_{MT}$ moves with constant velocity $\left(v_{x},v_{y}\right)$. Suppose that a linear frequency-modulated (LFM) signal is transmitted by SAR as:

(1) $$s\left(t\right)=\exp\left[j2\pi \left(f_{c}t+\frac{1}{2}\gamma t^{2}\right)\right]\mathrm{rect}\left(\frac{t}{T_{p}}\right)$$

where $f_{c}$,$\gamma =B/T_{p}$, *B*, $T_{p}$, and $\mathrm{rect}\left(\cdot \right)$ are the carrier frequency, the pulse chirp rate, the pulse bandwidth, the pulse duration of the LFM signal, and the rectangular window, respectively. After the demodulation, the base-band echoes of the $m^{\mathrm{th}}$ antenna element of the target $P_{MT}$ can be given by:

(2) $$\begin{array}{cc} s(t,\eta ,m)= & \omega_{a}\left(\eta \right)\exp\left[j\pi \gamma {\left(t-\frac{R_{0}\left(\eta ,x_{0},y_{0}\right)+R_{m}\left(\eta ,x_{0},y_{0}\right)}{c}\right)}^{2}\right]\\ & \times \exp\left[-j2\pi \frac{R_{0}\left(\eta ,x_{0},y_{0}\right)+R_{m}\left(\eta ,x_{0},y_{0}\right)}{\lambda }\right] \end{array}$$

where $\omega_{a}\left(\eta \right)$ represents the antenna pattern function in the azimuth direction approximated as $\omega_{a}\left(\eta \right)=\mathrm{rect}\left(\frac{\eta }{T_{s}}\right)$, $T_{s}$ is the synthetic aperture time, λ is the wavelength, and *c* is the light speed. $R_{0}\left(\eta ,x_{0},y_{0}\right)$ and $R_{m}\left(\eta ,x_{0},y_{0}\right)$ are the distance from $P_{MT}$ to the zeroth and $m^{\mathrm{th}}$ antenna element at η, respectively, which can be written as:

(3) $$\begin{array}{c} R_{m}\left(\eta ,x_{0},y_{0}\right)=\sqrt{{\left[\left(v_{p}\eta +\frac{1}{2}a\eta^{2}+md\right)-v_{x}\eta \right]}^{2}+{\left(y_{0}+v_{y}\eta \right)}^{2}+h^{2}}\\ m=0,1,\ldots ,M-1 \end{array}$$

After the range compression of Equation (2), the received signal can be given by:

(4) $$s_{rc}\left(t,\eta ,m\right)=T_{p}\mathrm{sinc}\left[B\left(t-\frac{R_{0m}\left(\eta ,x_{0},y_{0}\right)}{c}\right)\right]\mathrm{rect}\left(\frac{\eta }{T_{s}}\right)\exp\left[-j2\pi \frac{R_{0m}\left(\eta ,x_{0},y_{0}\right)}{\lambda }\right]$$

where $\mathrm{sinc}\left(x\right)=\frac{sin(\pi x)}{\pi x}$ and $R_{0m}\left(\eta ,x_{0},y_{0}\right)$ is the two-way range of the target $P_{MT}$ to the $m^{\mathrm{th}}$ antenna element. According to the equivalent phase center principle, the $m^{\mathrm{th}}$ receive antenna with m≠0 can be equalized to a virtual antenna that transmits and receives signals independently, lying in the center of the line from the transmit antenna to the $m^{\mathrm{th}}$ receive antenna [33,34]. The $m^{\mathrm{th}}$ virtual antenna is located at $\left(\frac{md}{2},0,h\right)$ when η=0. Therefore, $R_{0m}\left(\eta ,x_{0},y_{0}\right)$ can be approximated as:

(5) $$\begin{array}{c} R_{0m}\left(\eta ,x_{0},y_{0}\right)=R_{0}\left(\eta ,x_{0},y_{0}\right)+R_{m}\left(\eta ,x_{0},y_{0}\right)\\ \approx 2\sqrt{{\left[\left(v_{p}\eta +\frac{1}{2}a\eta^{2}+\frac{md}{2}\right)-v_{x}\eta \right]}^{2}+{\left(y_{0}+v_{y}\eta \right)}^{2}+h^{2}} \end{array}$$

## 3. Proposed GMTI Method for Multichannel Maneuvering SAR

### 3.1. Moving Target Detection and Range Velocity Estimation

#### 3.1.1. Moving Target Imaging with the BP Algorithm

The BP algorithm, which is applicable for an arbitrary platform trajectory, is adopted to reconstruct the static scene with high accuracy. Here, a BP imaging model is derived for an isolated point moving target $P_{MT}$ of the multichannel maneuvering SAR with fixed acceleration.

In the BP algorithm, a projection plane with height $h_{1}=0$ is adopted for scene reconstruction [35]. The correlation function in the time domain used for the azimuth focusing of the BP algorithm is:

(6) $$h\left(x,y,t,\eta ,m\right)=\omega \left(\eta ,x,y\right)\delta \left[\tau -\frac{D_{0m}\left(\eta ,x,y\right)}{c}\right]\exp\left[j\frac{2\pi }{\lambda }D_{0m}\left(\eta ,x,y\right)\right]$$

where $\omega \left(\eta ,x,y\right)$ is the window function and $D_{0m}\left(\eta ,x,y\right)$ is the two-way range of a pixel point $P_{ref}\left(x,y,0\right)$ that is close to the focus position of $P_{MT}$ in the projection plane, which can be given by:

(7) $$D_{0m}\left(\eta ,x,y\right)\approx 2\sqrt{{\left[\left(v_{p}\eta +\frac{1}{2}a\eta^{2}+\frac{md}{2}\right)-x\right]}^{2}+y^{2}+h^{2}}$$

Then, the BP response of the target $P_{MT}$ at point $P_{ref}\left(x,y,0\right)$ can be obtained by an integral as:

(8) $$\begin{array}{cc} I\left(x,y,m\right) & =\int h\left(x,y,t,\eta ,m\right)s_{rc}\left(t,\eta ,m\right)d\eta \\ & =T_{p}\int_{-\frac{T_{s}}{2}}^{\frac{T_{s}}{2}}\mathrm{sinc}\left\{B\left[-\frac{R_{0m}\left(\eta ,x_{0},y_{0}\right)}{c}+\frac{D_{0m}\left(\eta ,x,y\right)}{c}\right]\right\}\\ & \times \exp\left[-j\frac{2\pi }{\lambda }R_{0m}\left(\eta ,x_{0},y_{0}\right)+j\frac{2\pi }{\lambda }D_{0m}\left(\eta ,x,y\right)\right]d\eta \end{array}$$

Assume $P_{MT}$ can be focused in a single range cell in the image; thus, Equation (8) can be rewritten as:

(9) $$I\left(x,y,m\right)=T_{p}\mathrm{sinc}\left\{B\left[-\frac{R_{0m}\left(0,x_{0},y_{0}\right)}{c}+\frac{D_{0m}\left(0,x,y\right)}{c}\right]\right\}\int_{-\frac{T_{s}}{2}}^{\frac{T_{s}}{2}}\exp\left[j\varphi_{m}\left(\eta \right)\right]d\eta$$

The phase function in the integral of Equation (9) can be expressed as:

(10) $$\varphi_{m}\left(\eta \right)=\frac{2\pi }{\lambda }\left[D_{0m}\left(\eta ,x,y\right)-R_{0m}\left(\eta ,x_{0},y_{0}\right)\right]$$

The Taylor series expansion of the phase function $\varphi_{m}\left(\eta \right)$ is:

(11) $$\varphi_{m}\left(\eta \right)\approx \varphi_{m}\left(0\right)+\varphi_{m}^{{}^{\prime }}\left(0\right)\eta +\frac{1}{2}\varphi_{m}^{"}\left(0\right)\eta^{2}+\cdot \cdot \cdot$$

In order to simplify the analysis, the coefficients of the order higher than the second order of Equation (11) are omitted. Then, the BP response of the target $P_{MT}$ at point $\left(x,y,0\right)$ of the $m^{\mathrm{th}}$ receiving channel can be represented as:

(12) $$\begin{array}{cc} I\left(x,y,m\right) & =T_{p}\mathrm{sinc}\left\{B\left[\frac{D_{0m}\left(0,x,y\right)-R_{0m}\left(0,x_{0},y_{0}\right)}{c}\right]\right\}\\ & \times \exp\left[j\varphi_{m}\left(0\right)\right]\int_{-\infty }^{\infty }\mathrm{rect}\left(\frac{\eta }{T_{s}}\right)\exp\left\{j\left[\varphi_{m}^{{}^{\prime }}\left(0\right)\eta +\frac{1}{2}\varphi_{m}^{"}\left(0\right)\eta^{2}\right]\right\} \end{array}$$

Then, we obtain the BP imaging model of the target $P_{MT}$ by dividing Equation (12) into two cases according to the 2D velocity of the target $P_{MT}$.

- Case 1: the target $P_{MT}$ with its 2D velocity $v_{x}=0\;\&\&\;v_{y}=0$:
  (13) $$\begin{array}{cc} I\left(x,y,m\right)= & T_{p}T_{s}\mathrm{sinc}\left[2B\left(\frac{\sqrt{x^{2}+y^{2}+h^{2}}-\sqrt{h^{2}+y_{0}^{2}}}{c}\right)\right]\\ & \times \mathrm{sinc}\left[\frac{4T_{s}v_{p}x}{D_{00}\left(0,x,y\right)\lambda }\right]\exp\left[-j\frac{4\pi }{\lambda }\frac{xdm}{D_{00}\left(0,x,y\right)}\right] \end{array}$$
- Case 2: the target $P_{MT}$ with its 2D velocity $v_{x}\neq 0\;\left|\right|\;v_{y}\neq 0$:
  (14) $$\begin{array}{cc} I\left(x,y,m\right)\approx & A\left(x,y\right)\mathrm{sinc}\left[2B\left(\frac{\sqrt{x^{2}+y^{2}+h^{2}}-\sqrt{h^{2}+y_{0}^{2}}}{c}\right)\right]\\ & \times \mathrm{rect}\left[\frac{x+\frac{v_{y}y_{0}}{v_{p}}}{\frac{\left(2v_{p}v_{x}-ax-v_{x}^{2}-v_{y}^{2}\right)T_{s}}{v_{p}}}\right]\exp\left(-j\frac{4\pi }{\lambda }\frac{xdm}{D_{00}\left(0,x,y\right)}\right) \end{array}$$

The proof of Equations (13) and (14) can be found in Appendix A. Based on the BP imaging models of the target $P_{MT}$ given in Equations (13) and (14), we are able to analyze the properties of a moving target in the multichannel SAR image domain, including its position offset and the peak response, which can provide a theoretical support for the proposed method.

#### 3.1.2. Locate a Moving Target in the Image Domain

To analyze the peak response of a moving target in the multichannel SAR images, we should locate the moving target in the image domain at first. Define point $({\hat{x}}_{m},{\hat{y}}_{m}),m=0,1,2\ldots M-1$ as the focus position of the target $P_{MT}$ in the $m^{\mathrm{th}}$ receiving channel SAR image; $\left({\hat{x}}_{m},{\hat{y}}_{m}\right)$ is the position of the peak response with the maximum amplitude of the target $P_{MT}$ in the $I\left(x,y,m\right)$, which can be given by:

(15) $$\left({\hat{x}}_{m},{\hat{y}}_{m}\right)=\max_{\left(x,y\right)}\left|I\left(x,y,m\right)\right|,m=0,1,\ldots ,M-1$$

Since the focus positions of the moving target are all identical in the images of different receiving channels, their coordinates in SAR images of different channels are the same [35]. We use $\left(\hat{x},\hat{y}\right)$ to represent the focus positions of the moving target uniformly in the SAR images; therefore, we have:

(16) $$\left(\hat{x},\hat{y}\right)=\left({\hat{x}}_{0},{\hat{y}}_{0}\right)=\left({\hat{x}}_{1},{\hat{y}}_{1}\right)=\ldots =\left({\hat{x}}_{M-1},{\hat{y}}_{M-1}\right)$$

For Case 1 in Section 3.1.1, i.e., $v_{x}=0\;\&\&\;v_{y}=0$, it is shown from Equation (13) that the target can be focused as a 2D “peak” in the image domain, and its focus position is:

(17) $$\left\{\begin{array}{c} \hat{x}=0\\ \sqrt{{\hat{x}}^{2}+{\hat{y}}^{2}+h^{2}}=\sqrt{y_{0}^{2}+h^{2}} \end{array}\right.\Rightarrow \left\{\begin{array}{c} \hat{x}=0=x_{0}\\ \hat{y}=y_{0} \end{array}\right.$$

Equation (17) means if the target $P_{MT}$ has no velocity, i.e., it is a static target, its focus positions in the multichannel SAR images are its real position $\left(x_{0},y_{0}\right)$ with no shift.

On the other hand, for Case 2 in Section 3.1.1, i.e., $v_{x}\neq 0\;\left|\right|\;v_{y}\neq 0$, it can be seen from Equation (14) that due to the target motion, the moving target in the image domain is not a 2D peak, but is distributed in a single range unit and is “defocused” along the azimuth direction as a “straight line”. From Equations (14)–(16), it is easy to obtain the focus position of the moving target $P_{MT}$ as:

(18) $$\left\{\begin{array}{c} \hat{x}+\frac{v_{y}y_{0}}{v_{p}}=0\\ \sqrt{{\left(\frac{v_{y}y_{0}}{v_{p}}\right)}^{2}+{\hat{y}}^{2}+h^{2}}\approx \sqrt{y_{0}^{2}+h^{2}} \end{array}\right.\Rightarrow \left\{\begin{array}{c} \hat{x}=-\frac{v_{y}y_{0}}{v_{p}}\\ \hat{y}\approx y_{0} \end{array}\right.$$

**Figure 2:**
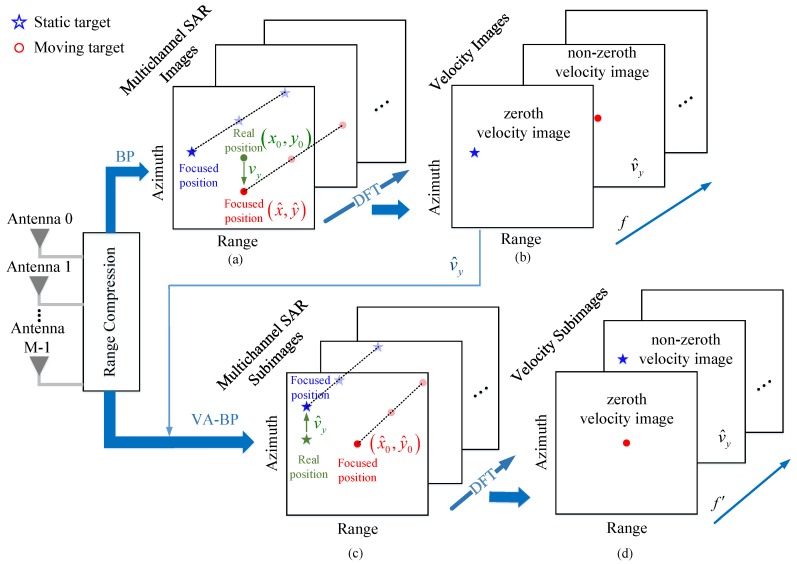
SAR images of different methods. (**a**) Multichannel SAR images via the BP algorithm. (**b**) Velocity images. (**c**) Multichannel SAR subimages via the velocity-aided (VA)-BP algorithm. (**d**) Velocity subimages.

Therefore, the azimuth offset and range offset relative to the static target in Equation (17) can be given for the moving target as:

(19) $$\left\{\begin{array}{c} \Delta x=\hat{x}-x_{0}=-\frac{v_{y}y_{0}}{v_{p}}\\ \Delta y=\hat{y}-y_{0}=0 \end{array}\right.$$

which means that the focus position of a moving has an azimuth shift and almost no range shift in the SAR image domain compared with its real position $\left(x_{0}=0,y_{0}\right)$ as sketched in Figure 2a. In addition, the azimuth shift of the moving target is related to its range velocity.

#### 3.1.3. Normalized Frequency of a Moving Target

In order to detect the moving target and estimate its range velocity for the multichannel maneuvering SAR, we should analyze the peak response of a moving target in the image domain. The peak response of $P_{MT}$ in the image domain can be presented as:

(20) $$I\left(\hat{x},\hat{y},m\right)=A\left(\hat{x},\hat{y}\right)\exp\left[-j\frac{4\pi }{\lambda }\frac{\hat{x}dm}{D_{00}\left(0,\hat{x},\hat{y}\right)}\right]=A_{0}\exp\left[j2\pi \frac{-\hat{x}dm}{\lambda \left[\frac{D_{00}\left(0,\hat{x},\hat{y}\right)}{2}\right]}\right]$$

where $A_{0}=A\left(\hat{x},\hat{y}\right)$ is a constant value that is unrelated to the position of the receiving channel.

For m=0,1,…,M−1, define the normalized frequency as [17]:

(21) $$f=\frac{-\hat{x}d}{\lambda \left[\frac{D_{00}\left(0,\hat{x},\hat{y}\right)}{2}\right]}$$

Substituting Equation (21) into Equation (20), we can get:

(22) $$I\left(\hat{x},\hat{y},m\right)=A_{0}\exp\left(j2\pi fm\right)$$

It is shown from Equations (17) and (21) that if $P_{MT}$ has no velocity, i.e., it is a static target, its normalized frequency f=0. It is shown from Equations (18) and (21) that if $P_{MT}$ is a moving target with $v_{y}\neq 0$, its normalized frequency f≠0.

#### 3.1.4. Moving Target Detection and Range Velocity Estimation

After the BP imaging process, *M* multichannel SAR images can be generated. The signal of the target along the multichannels in a given pixel in the SAR image can be expressed by Equation (22) as:

(23) $$\Gamma =A_{0}{\left[1,\exp\left(j2\pi f\right),\exp\left(j2\pi 2f\right),\cdots ,\exp\left(j2\pi (M-1)f\right)\right]}^{T}$$

where ${\left(\cdot \right)}^{T}$ denotes the matrix transpose operation. It is shown that the signal of the target along multichannels can be approximated as a sinusoid with a normalized frequency *f* and the number of channel *m*. Then, the clutter suppression can be achieved by the VSAR method [17,23,36]. The main steps of VSAR processing are listed as follows

- Step 1: For every pixel of *M* SAR images, the *M*-point DFT along the antenna array direction can be taken to transform *M* SAR images into velocity images.
- Step 2: The stationary clutter can be suppressed by replacing the zeroth velocity image by zero.
- Step 3: The *M*-point inverse DFT along the antenna array direction can be taken to transform velocity images back to SAR images.

Here, the moving target is separated from the clutter, and then, it is detected in one of the clutter-suppressed SAR image via the constant false alarm rate rule (CFAR). After the moving target is detected, from Equation (23), it can be seen that its normalized frequency *f* can be estimated via DFT of Γ in terms of *m*. According to [25], the accuracy of the frequency estimation is restricted by the velocity resolution determined by the array length in VSAR processing. Therefore, in order to improve the accuracy of frequency estimation, the zero-padded $N_{DFT}$-point DFT along the antenna array direction is taken in the pixel point of the moving target to obtain the interpolated velocity spectrum, and then, the normalized frequency of the moving target denoted as $\hat{f}$ is estimated by searching for the “peak” in the velocity spectrum [17]. Afterwards, the estimated value of the range velocity denoted as ${\hat{v}}_{y}$ can be calculated by Equations (18) and (21) as:

(24) $${\hat{v}}_{y}=\frac{\lambda v_{p}D_{00}\left(0,\hat{x},\hat{y}\right)}{2\hat{y}d}\hat{f}$$

Accordingly, the moving target $P_{MT}$ can be relocated by Equation (19) as:

(25) $$\left\{\begin{array}{c} {\hat{x}}_{0}=\hat{x}+\frac{{\hat{v}}_{y}\hat{y}}{v_{p}}\\ {\hat{y}}_{0}\approx \hat{y} \end{array}\right.$$

where $\left({\hat{x}}_{0},{\hat{y}}_{0}\right)$ is the estimated real position of the moving target in the SAR image.

### 3.2. Moving Target Azimuth Velocity Estimation, Refocusing, and Relocation

As for azimuth velocity, we adopt the velocity search method to obtain its estimated value and achieve the refocusing of the moving target at the same time. First, a subimage space that contains the estimated real position of a moving target obtained by Equation (25) is selected to refocus the moving target. Second, the estimated range velocity and the searched different azimuth velocities are utilized to get a series of multichannel SAR subimages by the VA-BP algorithm. On the one hand, the effect of the range velocity on the moving target including azimuth position offset and defocusing can be eliminated by the VA-BP algorithm, which means the moving target can be refocused on its real position, and its azimuth defocusing is only caused by the azimuth velocity. Once the azimuth velocity is searched accurately, the moving target will be focused well on its real position with the best quality. On the other hand, since the moving target and the static target have different normalized frequencies after the VA-BP algorithm, the VSAR technique can be applied again to suppress the stationary clutter in the multichannel SAR subimages. After the clutter is suppressed, subimages with different azimuth velocities that only contain moving targets can be obtained, and the azimuth velocity can be estimated by searching the subimage of the best focusing quality.

#### 3.2.1. Imaging a Moving Target with the VA-BP Algorithm

In the proposed VA-BP algorithm, the effect of 2D velocity on the Doppler phase of the moving target will be compensated in the imaging process, which is different from the BP algorithm. Here, a VA-BP imaging model is also derived for an isolated point moving target of the multichannel maneuvering SAR with fixed acceleration.

The correlation function constructed with the estimated range velocity ${\hat{v}}_{y}$ and the searched azimuth velocity ${\tilde{v}}_{x}$ can be given by:

(26) $$h\left(x,y,t,\eta ,m;{\hat{v}}_{y},{\tilde{v}}_{x}\right)=\omega \left(\eta ,x,y\right)\delta \left[\tau -\frac{{\tilde{D}}_{0m}\left(\eta ,x,y;{\hat{v}}_{y},{\tilde{v}}_{x}\right)}{c}\right]\exp\left[j\frac{2\pi }{\lambda }{\tilde{D}}_{0m}\left(\eta ,x,y;{\hat{v}}_{y},{\tilde{v}}_{x}\right)\right]$$

where ${\tilde{D}}_{0m}\left(\eta ,x,y;{\hat{v}}_{y},{\tilde{v}}_{x}\right)$ is the two-way range from the radar to the pixel point $P_{ref}^{\prime }\left(x,y,0\right)$ that is close to the focus position of $P_{MT}$ in a small projection plane with height $h_{2}=0$. For the sake of analysis, we assume that ${\tilde{v}}_{x}$ is very close to the real azimuth velocity $v_{x}$. Therefore, ${\tilde{D}}_{0m}\left(\eta ,x,y;{\hat{v}}_{y},{\tilde{v}}_{x}\right)$ can be written as:

(27) $${\tilde{D}}_{0m}\left(\eta ,x,y;{\hat{v}}_{y},{\tilde{v}}_{x}\right)\approx 2\sqrt{{\left[\left(v_{p}\eta +\frac{1}{2}a\eta^{2}+\frac{md}{2}\right)-\left(x+{\tilde{v}}_{x}\eta \right)\right]}^{2}+{\left(y+{\hat{v}}_{y}\eta \right)}^{2}+h^{2}}$$

The integral of the VA-BP algorithm can be represented as:

(28) $$\begin{array}{cc} I_{sub}\left(x,y,m;{\hat{v}}_{y},{\tilde{v}}_{x}\right) & =\int h\left(x,y,t,\eta ,m;{\hat{v}}_{y},{\tilde{v}}_{x}\right)s_{rc}\left(t,\eta ,m\right)d\eta \\ & =T_{p}\int_{-\frac{T_{s}}{2}}^{\frac{T_{s}}{2}}\mathrm{sinc}\left\{B\left[-\frac{R_{0m}\left(\eta ,x_{0},y_{0}\right)}{c}+\frac{{\tilde{D}}_{0m}\left(\eta ,x,y;{\hat{v}}_{y},{\tilde{v}}_{x}\right)}{c}\right]\right\}\\ & \exp\left[-j\frac{2\pi }{\lambda }R_{0m}\left(\eta ,x_{0},y_{0}\right)+j\frac{2\pi }{\lambda }{\tilde{D}}_{0m}\left(\eta ,x,y;{\hat{v}}_{y},{\tilde{v}}_{x}\right)\right]d\eta \end{array}$$

The phase function in the integral of Equation (28) is expressed as:

(29) $${\tilde{\varphi }}_{m}\left(\eta \right)=\frac{2\pi }{\lambda }\left[{\tilde{D}}_{0m}\left(\eta ,x,y;{\hat{v}}_{y},{\tilde{v}}_{x}\right)-R_{0m}\left(\eta ,x_{0},y_{0}\right)\right]$$

The integral of the VA-BP can be calculated in a similar way to the BP integral. Here, the calculation process is omitted, and we directly give the results as:

- Case 1: the target $P_{MT}$ with its 2D velocity $v_{x}=0\;\&\&\;v_{y}=0$:
  (30) $$\begin{array}{cc} I_{sub}\left(x,y,m;{\hat{v}}_{y},{\tilde{v}}_{x}\right)\approx & \tilde{A}\left(x,y\right)\mathrm{sinc}\left\{2B\left[\frac{\sqrt{x^{2}+h^{2}+y^{2}}-\sqrt{h^{2}+y_{0}^{2}}}{c}\right]\right\}\\ & \times \mathrm{rect}\left[\left(\frac{x-\left({\hat{v}}_{y}y\right)/\left(v_{p}-{\tilde{v}}_{x}\right)}{\left({\tilde{v}}_{x}^{2}-2v_{p}{\tilde{v}}_{x}-ax\right)T_{s}/\left({\tilde{v}}_{x}-v_{p}\right)}\right)\right]\exp\left[-j\frac{4\pi }{\lambda }\frac{xdm}{{\tilde{D}}_{00}\left(0,x,y;{\hat{v}}_{y},{\tilde{v}}_{x}\right)}\right] \end{array}$$
- Case 2: the target $P_{MT}$ with its 2D velocity $v_{x}\neq 0\;\left|\right|\;v_{y}\neq 0$:
  (31) $$\begin{array}{cc} I_{sub}\left(x,y,m;{\hat{v}}_{y},{\tilde{v}}_{x}\right)= & T_{p}T_{s}\mathrm{sinc}\left\{2B\left[\frac{\sqrt{x^{2}+h^{2}+y^{2}}-\sqrt{h^{2}+y_{0}^{2}}}{c}\right]\right\}\\ & \times \mathrm{sinc}\left[\frac{T_{s}}{\lambda }\frac{\left(-4xv_{p}\right)+\left(4y{\hat{v}}_{y}-4v_{y}y_{0}\right)}{{\tilde{D}}_{00}\left(0,x,y;{\hat{v}}_{y},{\tilde{v}}_{x}\right)}\right]\exp\left[-j\frac{4\pi }{\lambda }\frac{xdm}{{\tilde{D}}_{00}\left(0,x,y;{\hat{v}}_{y},{\tilde{v}}_{x}\right)}\right] \end{array}$$

where $\tilde{A}\left(x,y\right)$ in Equation (30) is also a complex signal as in Equation (14), which is expressed as:

(32) $$\tilde{A}\left(x,y\right)=T_{p}\sqrt{\frac{2\pi }{\varphi_{m}^{"}\left(0\right)}}\exp\left[-j\frac{\pi }{\lambda }\frac{1}{{\tilde{D}}_{00}\left(0,x,y;{\hat{v}}_{y},{\tilde{v}}_{x}\right)}\frac{{\left[2x\left({\tilde{v}}_{x}-v_{p}\right)+2{\hat{v}}_{y}y-{\tilde{v}}_{x}dm\right]}^{2}}{\left({\tilde{v}}_{x}^{2}-2v_{p}{\tilde{v}}_{x}-ax\right)}\right]\exp\left(j\frac{\pi }{4}\right)$$

From Equations (30) and (31), we can see that if the VA-BP algorithm is utilized to focus the illuminated scene, the static target will be defocused in the image domain, while the moving target will be focused as a 2D peak in the image domain, which is opposite the BP algorithm.

#### 3.2.2. Location and Peak Response Analysis of a Moving Target via the VA-BP Algorithm

Similarly, assume the focus position of the target $P_{MT}$ is $\left({\hat{x}}^{\prime },{\hat{y}}^{\prime }\right)$ in the multichannel SAR subimages by the VA-BP algorithm. For Case 1 in Section 3.2.1, i.e., $v_{x}=0\;\&\&\;v_{y}=0$, according to Equation (30), it can be seen that the focus position of the target in the image domain is:

(33) $$\left\{\begin{array}{c} {\hat{x}}^{\prime }-\frac{{\hat{v}}_{y}{\hat{y}}^{\prime }}{\left(v_{p}-{\tilde{v}}_{x}\right)}=0\\ \sqrt{{\hat{x}}^{\prime 2}+{\hat{y}}^{\prime 2}+h^{2}}-\sqrt{y_{0}^{2}+h^{2}}=0 \end{array}\right.\Rightarrow \left\{\begin{array}{c} {\hat{x}}^{\prime }=\frac{{\hat{v}}_{y}{\hat{y}}^{\prime }}{\left(v_{p}-{\tilde{v}}_{x}\right)}\\ {\hat{y}}^{\prime }\approx y_{0} \end{array}\right.$$

Its peak response in the $m^{\mathrm{th}}$ SAR image is:

(34) $$I_{sub}\left({\hat{x}}^{\prime },{\hat{y}}^{\prime },m;{\hat{v}}_{y},{\tilde{v}}_{x}\right)=A_{1}\exp\left[-j\frac{4\pi }{\lambda }\frac{{\hat{x}}^{\prime }dm}{{\tilde{D}}_{00}\left(0,{\hat{x}}^{\prime },{\hat{y}}^{\prime };{\hat{v}}_{y},{\tilde{v}}_{x}\right)}\right]$$

where $A_{1}=\tilde{A}\left({\hat{x}}^{\prime },{\hat{y}}^{\prime }\right)$ is also a constant value that is unrelated to the position of the receiving channel.

For Case 2 in Section 3.2.1, i.e., $v_{x}\neq 0\;\left|\right|\;v_{y}\neq 0$, according to Equation (31), it can be seen that the focus position of the target in the image domain is:

(35) $$\left\{\begin{array}{c} -4{\hat{x}}^{\prime }v_{p}+\left(4{\hat{y}}^{\prime }{\hat{v}}_{y}-4v_{y}y_{0}\right)=0\\ \sqrt{{\hat{x}}^{\prime 2}+{\hat{y}}^{\prime 2}+h^{2}}-\sqrt{y_{0}^{2}+h^{2}}=0 \end{array}\right.\Rightarrow \left\{\begin{array}{c} {\hat{x}}^{\prime }\approx {\hat{x}}_{0}\\ {\hat{y}}^{\prime }\approx {\hat{y}}_{0} \end{array}\right.$$

Its peak response in the $m^{\mathrm{th}}$ SAR image is:

(36) $$I_{sub}\left({\hat{x}}^{\prime },{\hat{y}}^{\prime },m;{\hat{v}}_{y},{\tilde{v}}_{x}\right)=T_{p}T_{s}\exp\left[-j\frac{4\pi }{\lambda }\frac{{\hat{x}}^{\prime }dm}{{\tilde{D}}_{00}\left(0,{\hat{x}}^{\prime },{\hat{y}}^{\prime };{\hat{v}}_{y},{\tilde{v}}_{x}\right)}\right]$$

It can be found from Equations (33) and (35) that if the target $P_{MT}$ has no velocity, i.e., it is a static target, the target will be shifted in the azimuth direction in the subimage, and if the target $P_{MT}$ is a moving target with $v_{y}\neq 0$, it will be focused well at its estimated real position $\left({\hat{x}}_{0},{\hat{y}}_{0}\right)$ via the VA-BP algorithm, as shown in Figure 2c.

#### 3.2.3. Normalized Frequency Analysis of a Moving Target via the VA-BP Algorithm

For m=0,1,…,M−1, the normalized frequency of the target $P_{MT}$ is:

(37) $$f^{\prime }=\frac{2}{\lambda }\frac{-{\hat{x}}^{\prime }d}{{\tilde{D}}_{00}\left(0,{\hat{x}}^{\prime },{\hat{y}}^{\prime };{\hat{v}}_{y},{\tilde{v}}_{x}\right)}$$

According to Equations (33) and (34), we can see, if $P_{MT}$ is a static target, its normalized frequency is:

(38) $$f^{\prime }=\frac{2}{\lambda }\frac{-{\hat{v}}_{y}{\hat{y}}^{\prime }d}{{\tilde{D}}_{00}\left(0,{\hat{x}}^{\prime },{\hat{y}}^{\prime };{\hat{v}}_{y},{\tilde{v}}_{x}\right)}\neq 0$$

On the other hand, from Equations (35) and (36), we can see that if $P_{MT}$ is a moving target, its normalized frequency is:

(39) $$f^{\prime }=\frac{2}{\lambda }\frac{-{\hat{x}}_{0}d}{{\tilde{D}}_{00}\left(0,{\hat{x}}^{\prime },{\hat{y}}^{\prime };{\hat{v}}_{y},{\tilde{v}}_{x}\right))}\approx \frac{2}{\lambda }\frac{-x_{0}d}{{\tilde{D}}_{00}\left(0,{\hat{x}}^{\prime },{\hat{y}}^{\prime };{\hat{v}}_{y},{\tilde{v}}_{x}\right))}=0$$

#### 3.2.4. Moving Target Azimuth Velocity Estimation, Refocusing, and Relocation

For the estimated range velocity ${\hat{v}}_{y}$ and each searched azimuth velocity ${\tilde{v}}_{x}$, *M* multichannel SAR subimages can be obtained by the VA-BP algorithm, which are visualized in Figure 2c. Then, the *M*-point DFT operation is applied in every pixel of the *M* multichannel SAR subimages along the antenna array direction, and the static clutter is located in one of the no-zeroth velocity subimages, while the moving target is located in the zeroth velocity subimage according to Equations (38) and (39) as shown in Figure 2d. As a result, the static clutter is suppressed by VSAR processing, i.e., replacing the non-zeroth velocity subimages by zero. Then, the *M*-point inverse DFT along the antenna array direction is taken to transform velocity subimages back to SAR images. Therefore, the moving target is separated from the stationary clutter in the multichannel SAR subimages.

After the stationary clutter is suppressed, each subimage of the *M* channels only contains the moving target; the closer ${\tilde{v}}_{x}$ is approaching $v_{x}$, the higher quality the subimage has. The entropy can well represent the quality of an image [37,38]. Therefore, after the subimages $I_{sub}\left(p,q,0;{\hat{v}}_{y},{\tilde{v}}_{x}\right)$ under different ${\tilde{v}}_{x}$ are obtained (here, we use the subimage of the zeroth receiving channel, i.e., m=0), we build the relation graph between ${\tilde{v}}_{x}$ and the entropy of the subimage. The more accurate ${\tilde{v}}_{x}$ is to $v_{x}$, the smaller the entropy of the subimage is.

The entropy of a subimage is defined as:

(40) $$E\left({\tilde{v}}_{x}\right)=-\sum_{p,q}c_{pq}\ln c_{pq},p=1,\ldots N_{x};q=1,\ldots N_{y}$$

(41) $$c_{pq}=\frac{{\left|I_{sub}\left(p,q,0;{\hat{v}}_{y},{\tilde{v}}_{x}\right)\right|}^{2}}{\sum_{p=1}^{N_{x}}\sum_{q=1}^{N_{y}}{\left|I_{sub}\left(p,q,0;{\hat{v}}_{y},{\tilde{v}}_{x}\right)\right|}^{2}}$$

where $N_{x}\times N_{y}$ is the data size of subimage $I_{sub}$.

By searching the subimage with the minimum entropy, the azimuth velocity can be estimated as:

(42) $${\hat{v}}_{x}=\min\underset{{\tilde{v}}_{x}}{\arg}E\left({\tilde{v}}_{x}\right)$$

Once the estimated value of the azimuth velocity ${\hat{v}}_{x}$ is obtained, the clutter-suppressed subimage corresponding to ${\hat{v}}_{x}$ is that with the best refocusing quality of the moving target. since the 2D velocity of the moving target has been compensated in the VA-BP imaging process, the moving target will be refocused well on its true position in the image domain.

The VSAR technique can also obtain the corrected VSAR image such that azimuth displacement and defocusing of moving targets are both corrected. In [30], the multichannel SAR images were transformed into velocity images through a DFT operation, and the PGA algorithm was utilized to correct the defocussing of the moving targets in the velocity images. Then, the final corrected VSAR image was obtained by azimuthally shifting these velocity images and summing them together. However, in this paper, since the VA-BP algorithm is used to correct the defocussing of the moving targets, we use another method to obtain the final corrected VSAR image. First, the velocity images of stationary clutter and moving targets are obtained, respectively. Then, they are summed non-coherently to combine the final corrected VSAR image.

The steps to obtain the stationary (DC) velocity image are as follows

- Step 1: The BP algorithm with the correlation function of the stationary target is used to obtain *M* SAR images. Here, *M* is the number of receiving channels.
- Step 2: Perform *M*-point DFT along the antenna array direction to get the *M* velocity images.
- Step 3: Take out the zeroth velocity image, i.e., the DC velocity image.

The steps to obtain the corrected velocity image of each moving target are as follows:

- Step 1: The VA-BP algorithm with the correlation function of the moving target is used to obtain *M* SAR images.
- Step 2: Perform *M*-point DFT along the antenna array direction to get *M* velocity images. Here, the moving target is in the zeroth velocity image, and the azimuth displacement and defocusing of the moving target is corrected.
- Step 3: Take out the zeroth velocity image, i.e., the velocity image of the moving target with both azimuth displacement and defocusing corrected.

Then, the corrected VSAR image is obtained by non-coherently summing the DC image and the corrected velocity images of the moving targets, where the moving targets are all refocused and relocated at their true positions.

**Figure 3:**
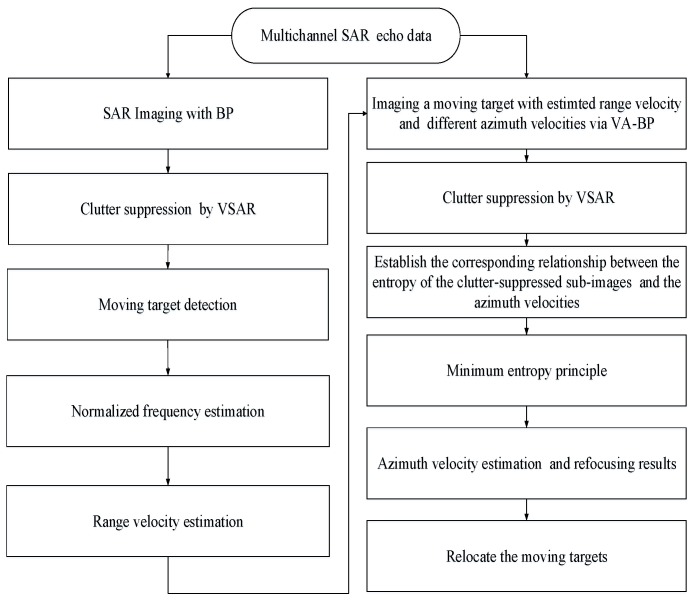
Flowchart of the proposed multichannel maneuvering SAR ground moving target indication (GMTI) method.

The flowchart of the proposed ground moving target 2D velocity estimation, refocusing and relocation method for multichannel maneuvering SAR is shown in Figure 3.

## 4. Numerical Experiments and Performance Analysis

In this section, SAR-GMTI simulation experiments on a maneuvering platform are conducted to demonstrate the effectiveness of the proposed method. Radar echoes of the static scene were acquired by an airborne multichannel SAR with the simulation parameters shown in Table 1. The static scene was intercepted from one real SAR image to get close to the actual environment, whose amplitude approximately obeyed a Rayleigh distribution. Six moving targets labeled as T1, T2, T3, T4, T5, and T6 were added into the echoes. The position and motion parameters of the moving targets are shown in Table 2, where T1, T2, and T3 are the point moving targets and T4, T5, and T6 are the extended moving targets. The SCR of the moving targets were all set as −5 dB in the image domain.

### 4.1. Moving Target Detection, 2D Velocity Estimation, and Refocusing

**Figure 4:**
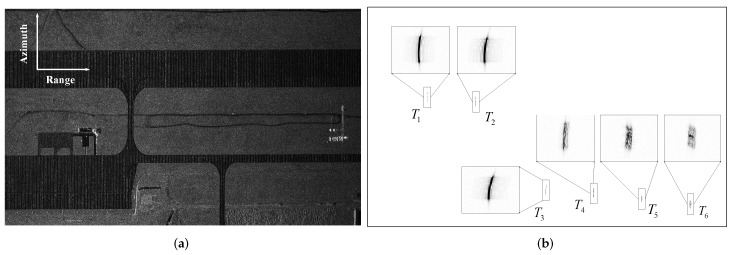
Moving targets detected in the SAR image: (**a**) the zeroth-channel SAR image without clutter suppression; (**b**) the zeroth-channel SAR image after clutter suppression and the detection results.

Multichannel SAR images were formed via BP algorithm. The zeroth receiving channel SAR image without suppressing the stationary clutter is displayed in Figure 4a, and it is difficult to detect the moving target in the image due to the strong background clutter. Therefore, the VSAR processing presented in Section 3.1.4 was utilized to suppress the stationary clutter. The zeroth channel SAR image after clutter suppression is shown in Figure 4b, where six moving targets marked with rectangles were all detected by the CFAR detector.

**Figure 5:**
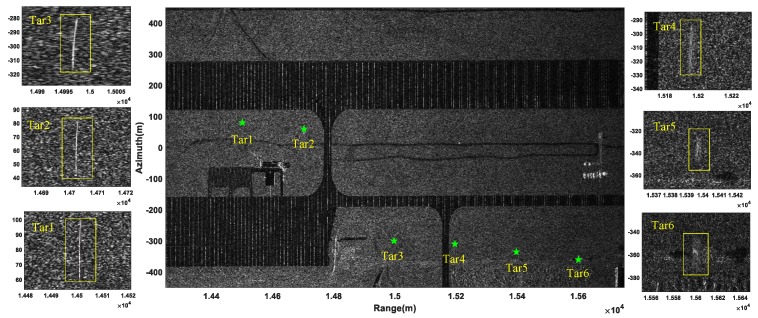
BP-VSAR image.

**Figure 6:**
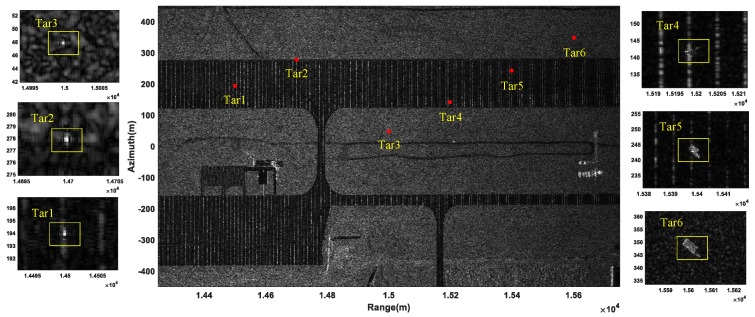
Corrected VSAR image.

After taking a DFT operation on each pixel over the eight SAR images obtained by the BP algorithm, eight velocity images were generated. The BP-VSAR image, which was obtained by non-coherently summing these eight velocity images, is shown in Figure 5, where the moving targets and the corresponding zoomed-in results are marked with green pentagrams and rectangles, respectively. We can see that the six moving targets were all defocused and shifted from their real positions. On the other hand, the corrected VSAR image is shown in Figure 6, where the moving targets and the corresponding zoomed-in results are marked with red point and the rectangles, respectively. Compared with Figure 5 and the position parameters of the moving targets shown in Table 2, it can be seen that the six moving targets were all refocused well and shifted back towards their actual initial positions.

The 2D velocity estimation results are presented in Table 2. It can be seen that the 2D velocities of six moving targets were all well estimated by the proposed method, with absolute errors smaller than 0.1 m/s.

**Figure 7:**
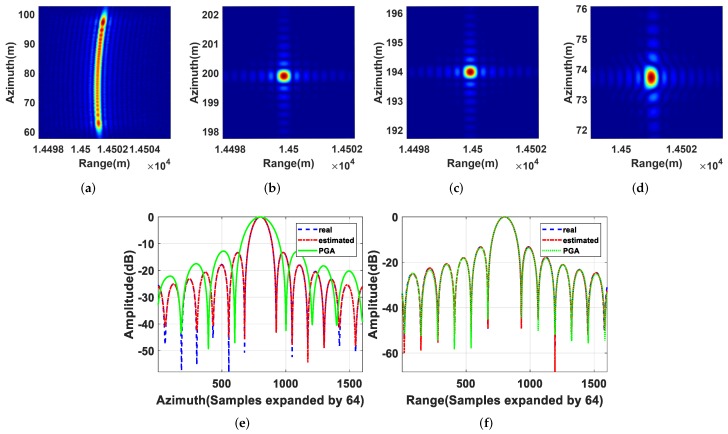
Refocusing results of T1: (**a**) defocusing result; (**b**) refocusing result using the real 2D velocity; (**c**) refocusing result using the estimated 2D velocity; (**d**) refocusing result using the phase gradient autofocus (PGA) algorithm; (**e**) azimuth profiles of T1 in (**b**–**d**); (**f**) range profiles of T1 in (**b**–**d**).

We took T1 as an example to illustrate the refocusing results of three point moving targets. Its defocusing result, refocusing result using the real 2D velocity, refocusing result using the estimated 2D velocity, and refocusing result using the PGA method are shown in Figure 7a–d, where the vertical axis represents the azimuth position coordinate and the horizontal axis represents the range position coordinate. Combined with the position parameter of T1 shown in Table 2, we can see, when the moving target was imaged using the parameter of the static target, it was defocused and shifted in the image domain. Besides, there was a range migration of the moving target in the image domain. When the moving target was refocused using the real 2D velocity via VA-BP, its range migration and azimuth defocusing caused by 2D velocity were eliminated in the imaging process, and it was focused well in its real position. On the other hand, when the estimated 2D velocity was used to refocus the moving target via VA-BP, it could be focused as well as the refocusing result using the real 2D velocity, except that the refocusing position was slightly shifted because of the error of the 2D velocity estimation. To further illustrate the effectiveness of the proposed refocusing method, we compared it with the PGA algorithm [30]. It can be obviously seen that the proposed refocusing method was superior to PGA algorithm, and there was a slight azimuth defocussing of the moving target using the PGA algorithm due to the fact that the PGA algorithm ignored the effects of the range migration of the moving target in the image domain.

Furthermore, the azimuth profiles and range profiles of Figure 7b–d are shown in Figure 7e,f, and the measured image quality parameters of three point moving targets are shown in Table 3, including the impulse response width (IRW) broadening, the left peak sidelobe ratio (PSLRL), and the right PSLR (PSLRR), which also proves the above conclusion quantitatively.

**Figure 8:**
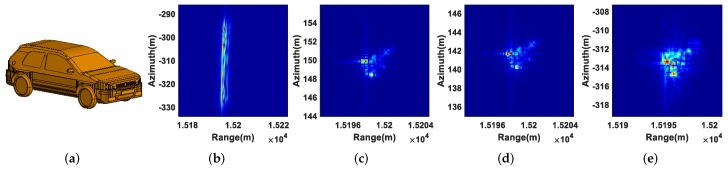
Refocusing results of T4: (**a**) shape of T4; (**b**) defocusing result; (**c**) refocusing result using the real 2D velocity; (**d**) refocusing result using the estimated 2D velocity; (**e**) refocusing result using the PGA algorithm.

**Figure 9:**
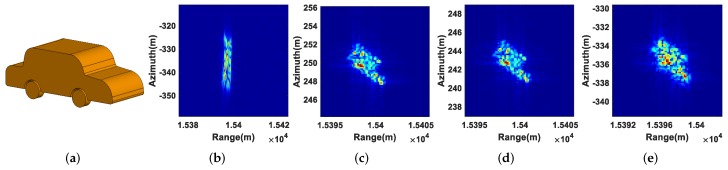
Refocusing results of T5: (**a**) shape of T5; (**b**) defocusing result; (**c**) refocusing result using the real 2D velocity; (**d**) refocusing result using the estimated 2D velocity; (**e**) refocusing result using the PGA algorithm.

**Figure 10:**
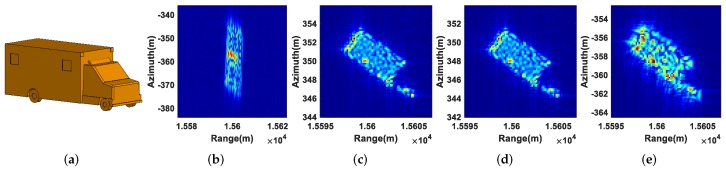
Refocusing results of T6: (**a**) shape of T6; (**b**) Defocusing result; (**c**) refocusing result using the real 2D velocity; (**d**) refocusing result using the estimated 2D velocity; (**e**) refocusing result using the PGA algorithm.

As for the extended moving targets T4, T5, and T6, their shapes, defocusing results, refocusing results using the real 2D velocity, refocusing results using the estimated 2D velocity, and refocusing results using the PGA algorithm are displayed in Figure 8, Figure 9 and Figure 10, from which we can come to the same conclusion as the refocusing results of the point moving targets.

### 4.2. Clutter Suppression Performance Analysis

**Figure 11:**
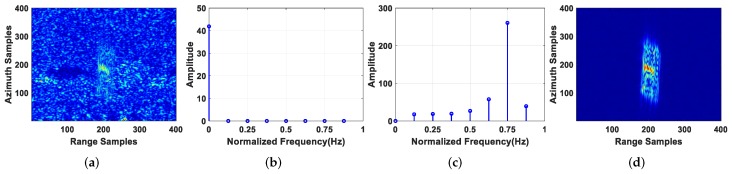
Imaging result of T6 via BP algorithm: (**a**) subimage of T6 before clutter suppression; (**b**) velocity spectrum of a static target in the subimage; (**c**) velocity spectrum of T6; (**d**) subimage of T6 after clutter suppression.

**Figure 12:**
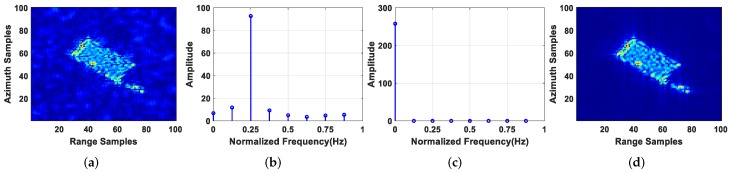
Imaging result of T6 via VA-BP algorithm: (**a**) subimage of T6 before clutter suppression; (**b**) velocity spectrum of a static target in the subimage; (**c**) velocity spectrum of T6; (**d**) subimage of T6 after clutter suppression.

Figure 11 shows the result of clutter suppression of T6, which is representative of the analysis. From Figure 11b,c, it can be seen that the normalized frequency of a static target was near 0 Hz, while that of a moving target was far from 0 Hz via the BP algorithm. Therefore, the clutter can be suppressed by the VSAR method, i.e., replacing the zeroth velocity image by zero. The subimages of T6 before and after clutter suppression are shown in Figure 11a,d. As can be seen, the clutter can be well suppressed via the BP-VSAR method. From Figure 12b,c, it can be seen that the normalized frequency of a static target is far from 0Hz while that of a moving target was near 0 Hz via the VA-BP algorithm, which was opposite the BP algorithm and consistent with Equations (38) and (39). Therefore, after the VA-BP algorithm, the VSAR method was used to suppress the clutter, i.e., by replacing the non-zeroth velocity images by zero. The subimages of T6 before and after clutter suppression via the VA-BP-VSAR method are shown in Figure 12a,d. We can see clearly that the clutter was suppressed greatly.

Table 4 gives six moving targets’ SCR improvement by the BP-VSAR and VA-BP-VSAR methods, where SCRin represents the input SCR, SCRout represents the output SCR, and SCRIm represents the SCR improvement. As can be seen, the SCR can both be improved greater than 22 dB, which shows that the BP-VSAR and VA-BP-VSAR methods can both acquire good clutter suppression results.

### 4.3. 2D Velocity Estimation Accuracy Analysis

**Figure 13:**
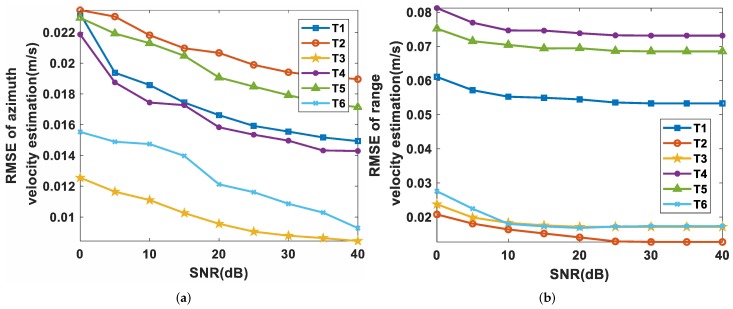
Performance analysis of 2D velocity estimation: (**a**) the RMS error of azimuth velocity estimation versus SNR; (**b**) the RMS error of range velocity estimation versus SNR.

The 2D velocity estimation accuracy was further investigated via 5000 Monte Carlo trials that were performed under different signal-to-noise (SNR) conditions. The root-mean-squared (RMS) errors of ${\hat{v}}_{x}$ and ${\hat{v}}_{y}$ were measured to indicate the estimation accuracy. As shown in Figure 13, the accuracy of 2D velocity estimation increased with the increase of SNR. The RMS errors of ${\hat{v}}_{x}$ and ${\hat{v}}_{y}$ were less than 0.03 m/s and 0.1 m/s for SNRs larger than 0 dB, and the RMS errors were almost the same for SNRs larger than 20 dB.

## 5. Conclusions

In this paper, a moving target 2D velocity estimation and refocusing method based on the BP algorithm and VSAR method was proposed for multichannel maneuvering SAR with fixed acceleration. First, BP was utilized to image the multichannel raw echoes. Second, the VSAR method was applied to suppress the stationary clutter and estimate the range velocity of the moving target. As for azimuth velocity estimation and refocusing, we used the velocity search method to accomplish them. The estimated range velocity and the searched azimuth velocity was used to construct the time domain correlation function for refocusing the moving target via VA-BP algorithm. Then, the stationary clutter of the subimages was suppressed again by the VSAR method. Finally, the azimuth velocity of the moving target was obtained by searching the subimage with the best focusing quality measured by minimum entropy. The proposed method provided a novel solution for moving target 2D velocity estimation and refocusing in a multichannel maneuvering SAR. Numerical simulation results showed that the proposed method can acquire high accuracy in 2D velocity estimation and refocusing for both point-like moving targets and the extended moving targets. The absolute errors of 2D velocity estimation were smaller than 0.1 m/s. The PSLRs of the point moving targets were smaller than −12.12 dB, and their IRW broadening was smaller than 1%, which further verified the effectiveness of the proposed method. 

## Figures and Tables

**Table 1:** Simulation parameters.

Parameter	Value	Parameter	Value	Parameter	Value
Wavelength	0.03 m	Range bandwidth	500 MHz	Pulse repetition frequency	1300
Platform altitude	3 km	Platform velocity	150 m/s	Platform acceleration	1 $m/s^{2}$
Aperture time	about 5 s	Number of antennas	8	Antenna spacing	0.5 m
Resolution cell	0.3 m × 0.3 m	Near range	15 km	Clutter-to-noise-ratio	20 dB

**Table 2:** 2D velocity estimation results. T, target.

		Position	Value	Velocity	True Value	Estimated Value	Absolute Error
Point Moving Targets	T1	*x* (m)	200	$v_{x}$ (m/s)	3.5	3.52	0.02
		*y* (m)	14,500	$v_{y}$ (m/s)	1.3	1.24	0.06
	T2	*x* (m)	280	$v_{x}$ (m/s)	3	2.98	0.02
		*y* (m)	14700	$v_{y}$ (m/s)	2.3	2.28	0.02
	T3	*x* (m)	50	$v_{x}$ (m/s)	2.5	2.51	0.01
		*y* (m)	15,000	$v_{y}$ (m/s)	3.5	3.48	0.02
Extended Moving Targets	T4	*x* (m)	150	$v_{x}$ (m/s)	2	2.02	0.02
		*y* (m)	15,200	$v_{y}$ (m/s)	4.6	4.52	0.08
	T5	*x* (m)	250	$v_{x}$ (m/s)	−4	−3.98	0.02
		*y* (m)	15,400	$v_{y}$ (m/s)	5.7	5.63	0.07
	T6	*x* (m)	350	$v_{x}$ (m/s)	−5	−4.99	0.01
		*y* (m)	15,600	$v_{y}$ (m/s)	6.8	6.78	0.02

**Table 3:** Measured image quality parameters. PSLRL, left peak sidelobe ratio; PSLRR, right PSLR; IRW, impulse response width.

		Proposed Refocusing Method						PGA Method		
		Real 2D Velocity			Estimated 2D Velocity					
		PSLRL (dB)	PSLRR (dB)	IRW Broad.	PSLRL (dB)	PSLRR (dB)	IRW Broad.	PSLRL (dB)	PSLRR (dB)	IRW Broad.
T1	Azimuth	−13.35	−13.19	0%	−13.31	−13.26	0%	−12.7	−12.9	62%
	Range	−13.24	−13.2	0%	−13.27	−13.23	0%	−13.6	−13.6	0.3%
T2	Azimuth	−13.29	−13.28	0%	−12.1	−12.3	1%	−14.83	−14.04	81.9%
	Range	−13.29	−13.31	0%	−13.34	−13.29	0.2%	−13.91	−13.87	0.3%
T3	Azimuth	−13.34	−13.22	0%	−13.31	−13.22	0.2%	−13.75	−13.14	123%
	Range	−13.38	−13.32	0%	−13.29	−13.35	0%	−14.31	−14.36	0.3%

**Table 4:** Signal-to-clutter ratio (SCR) improvement of BP-VSAR and VA-BP-VSAR methods

	BP-VSAR	SCRin (dB)	SCRout (dB)	SCRIm (dB)	VA-BP-VSAR	SCRin (dB)	SCRout (dB)	SCRIm (dB)
Point Moving Targets	T1	−5	22	27	T1	−1	21	22
	T2	−5	21	26	T2	−2	26	28
	T3	−5	21	26	T3	−2	25	27
Extended Moving Targets	T4	−5	22	27	T4	−1	27	28
	T5	−5	21	26	T5	−4	22	26
	T6	−5	19	24	T6	−3	21	24

## Appendix A. Proof of Equation (12)

Since $\left[D_{0m}\left(0,x,y\right)-R_{0m}\left(0,x_{0},y_{0}\right)\right]\approx \left[D_{00}\left(0,x,y\right)-R_{00}\left(0,x_{0},y_{0}\right)\right]$, the BP integral of the target $P_{MT}$ can be rewritten as:

(A1) $$\begin{array}{cc} I\left(x,y,m\right) & =T_{p}\mathrm{sinc}\left\{B\left[\frac{D_{00}\left(0,x,y\right)-R_{00}\left(0,x_{0},y_{0}\right)}{c}\right]\right\}\\ & \times \exp\left(j\varphi_{m}\left(0\right)\right)\int_{-\infty }^{\infty }\mathrm{rect}\left(\frac{\eta }{T_{s}}\right)\exp\left\{j\left[\varphi_{m}^{\prime }\left(0\right)\eta +\frac{1}{2}\varphi_{m}^{"}\left(0\right)\eta^{2}\right]\right\}d\eta \end{array}$$

The Taylor coefficients of $\varphi_{m}\left(\eta \right)$ can be derived as:

(A2) $$\varphi_{m}\left(0\right)\approx \frac{4\pi }{\lambda }\left[-\frac{xdm}{D_{00}\left(0,x,y\right)}\right]$$

(A3) $$\varphi_{m}^{\prime }\left(0\right)\approx \frac{2\pi }{\lambda }\left[\frac{-4v_{p}x-4v_{y}y_{0}+2dmv_{x}}{D_{00}\left(0,x,y\right)}\right]$$

(A4) $$\varphi_{m}^{"}\left(0\right)\approx \frac{2\pi }{\lambda }\left[\frac{8v_{p}v_{x}-4ax-4v_{x}^{2}-4v_{y}^{2}}{D_{00}\left(0,x,y\right)}\right]$$

Then, we obtain the BP imaging model of the target $P_{MT}$ by dividing (A1) into two cases according to the 2D velocity of the target $P_{MT}$.

- Case 1: the target $P_{MT}$ with its 2D velocity $v_{x}=0\;\&\&\;v_{y}=0$:
  If $v_{x}=0\;\&\&\;v_{y}=0$, i.e., $P_{MT}$ is a point static target, the phase function $\varphi_{m}\left(\eta \right)$ can be approximated by its first-order Taylor expansion as [39]:
  (A5) $$\varphi_{m}\left(\eta \right)\approx \varphi_{m}\left(0\right)+\varphi_{m}^{\prime }\left(0\right)\eta$$
  The result of the BP integral is:
  (A6) $$\begin{array}{cc} I\left(x,y,m\right)= & T_{p}T_{s}\mathrm{sinc}\left\{B\left[\frac{D_{00}\left(0,x,y\right)-R_{00}\left(0,x_{0},y_{0}\right)}{c}\right]\right\}\\ & \mathrm{sinc}\left[\frac{4T_{s}v_{p}f_{c}x}{D_{00}\left(0,x,y\right)c}\right]\exp\left(-j\frac{4\pi }{\lambda }\frac{xdm}{D_{00}\left(0,x,y\right)}\right) \end{array}$$
- Case 2: the target $P_{MT}$ with its 2D velocity $v_{x}\neq 0\;\left|\right|\;v_{y}\neq 0$:
  If $v_{x}\neq 0\;\left|\right|\;v_{y}\neq 0$, i.e., $P_{MT}$ is a point moving target, the integral in (A1) is an integral of the LFM signal whose time-bandwidth product (TBP) usually satisfies the stationary phase principle (SPP) approximation [40]. Then, the response could be calculated based on the SPP as:
  (A7) $$\begin{array}{cc} I\left(x,y,m\right)\approx & A\left(x,y\right)\mathrm{sinc}\left[2B\left(\frac{\sqrt{x^{2}+y^{2}+h^{2}}-\sqrt{h^{2}+y_{0}^{2}}}{c}\right)\right]\\ & \times \mathrm{rect}\left[\frac{x+\frac{v_{y}y_{0}}{v_{p}}}{\frac{\left(2v_{p}v_{x}-ax-v_{x}^{2}-v_{y}^{2}\right)T_{s}}{v_{p}}}\right]\exp\left(-j\frac{4\pi }{\lambda }\frac{xdm}{D_{00}\left(0,x,y\right)}\right) \end{array}$$
  where $A\left(x,y\right)$ is a complex signal that is represented as:
  (A8) $$A\left(x,y\right)=T_{p}\sqrt{\frac{2\pi }{\varphi_{m}^{"}\left(0\right)}}\exp\left(-j\frac{\pi }{\lambda }\frac{1}{D_{00}\left(0,x,y\right)}\frac{{\left(dmv_{x}-2v_{p}x-2v_{y}y_{0}\right)}^{2}}{\left(2v_{p}v_{x}-ax-v_{x}^{2}-v_{y}^{2}\right)}\right)\exp\left(j\frac{\pi }{4}\right)$$
